# Nephrogenic Adenoma of the Urinary Bladder: A Review of the Literature

**DOI:** 10.1155/2015/704982

**Published:** 2015-02-02

**Authors:** Anthony Kodzo-Grey Venyo

**Affiliations:** Department of Urology, North Manchester General Hospital, Delaunays Road, Manchester, UK

## Abstract

*Background*. Nephrogenic adenoma of the urinary bladder (NAUB) is a rare lesion associated with nonspecific symptoms and could inadvertently be misdiagnosed. *Aim*. To review the literature. *Methods*. Various internet search engines were used.* Results*. NAUB is a benign tubular and papillary lesion of the bladder, is more common in men and adults, and has been associated with chronic inflammation/irritation, previous bladder surgery, diverticula, renal transplantation, and intravesical BCG; recurrences and malignant transformations have been reported. Differential diagnoses include clear cell adenocarcinoma, endocervicosis, papillary urothelial carcinoma, prostatic adenocarcinoma of bladder, and nested variant of urothelial carcinoma; most NAUBs have both surface papillary and submucosal tubular components; both the papillae and tubules tend to be lined by a single layer of mitotically inactive bland cells which have pale to clear cytoplasm. Diagnosis may be established by using immunohistochemistry (positive staining with racemase; PAX2; keratins stain positive with fibromyxoid variant), electron microscopy, DNA analysis, and cytological studies. *Treatment*. Endoscopic resection is the treatment but recurrences including sporadic malignant transformation have been reported. *Conclusions*. There is no consensus on best treatment. A multicentre study is required to identify the treatment that would reduce the recurrence rate, taking into consideration that intravesical BCG is associated with NAUB.

## 1. Introduction

Nephrogenic adenoma of the urinary bladder is a rare lesion which could easily be confused with or could easily be misdiagnosed as a number of malignant lesions of the urinary bladder. Nephrognic adenoma of the urinary bladder may either be associated with or it may be induced/triggered by a variety of inflammatory insults to the urinary bladder; some of which include: recurrent urinary tract infections, recurrent urinary tract calculi, intravesical therapy, “diverticula of the urinary bladder”, kidney transplantation, foreign bodies, radiotherapy, chemical agents and a number of irritative factors [1]. Mostofi in 1954 suggested that the urinary bladder epithelium had the ability to transform into several morphologic types under appropriate stimulation and also stated that squamous and glandular metaplasia of the urothelium is observed frequently associated with chronic infection [1].

Nephrogenic adenoma (nephrogenic metaplasia) is more common in the male in comparison with female with a male to female ratio of 2 : 1, and it has been reported to occur in a large range of age groups ranging from 4 years to 81 years [2]. Nephrogenic adenoma is more common in adults but it has been reported that about 10% of nephrogenic adenomas affect children [2]. There is some uncertainty about the origin of nephrogenic adenoma of the urinary bladder but a number of postulates exist regarding the pathogenesis of the disease and some of these include the following: it may be a metaplastic lesion; it may emanate embryonic tissue; it may be a metaplasia that occasionally coexists with multifocal urothelial carcinoma [3–5]; it may originate from embryonic mesonephroid tissue; a large number of reports had documented that cases of nephrogenic adenoma of the urinary bladder had emanated from urothelial injury pursuant to previous surgery or long-term inflammation; immunosuppressive therapy in kidney transplant patients and intravesical instillations of Bacillus Calmette-Guérin have also been linked with nephrogenic adenoma of the urinary bladder. The ensuing review paper on nephrogenic adenoma of the urinary bladder is divided into two parts: (A) overview and (B) miscellaneous narrations from some of the reported cases and case series of nephrogenic adenoma of the urinary bladder.

## 2. Methods

Various internet search engines including PUBMED were used to identify literature on nephrogenic adenoma of the urinary bladder which illustrate various aspects of nephrogenic adenoma of the urinary bladder. The key words used included nephrogenic adenoma of urinary bladder and nephrogenic metaplasia of urinary bladder. Seventy-four documents relating to nephrogenic adenoma of the urinary bladder were identified to be suitable for documenting the definition, presentation, investigation, and management as well as outcome following treatment for nephrogenic adenoma of the urinary bladder.

## 3. Results/Literature Review

### 3.1. Overview

#### 3.1.1. Definition

Nephrogenic adenoma of the urinary bladder is defined as a metaplastic change in the urinary bladder with papillary or cryptic structures which are composed of small hollow tubules similar to mesonephric tubules, which are usually lined by a single layer of bland cuboidal or hobnail cells, surrounding eosinophilic or basophilic secretions [7].

#### 3.1.2. Alternative Terminology

Nephrogenic adenoma has also been referred to as mesonephric adenoma/metaplasia, adenomatoid tumour, and adenomatoid metaplasia [7].

#### 3.1.3. Epidemiology

- Nephrogenic adenoma of the urinary bladder occurs in adults and rarely in children [8].
- Nephrogenic adenoma of the urinary bladder occurs more commonly in males in comparison with females (2/3 in males) [7].

#### 3.1.4. Sites of the Urinary Tract Affected

- Nephrogenic adenoma tends to affect the urinary bladder, urethra, ureter, and renal pelvis in decreasing frequency [7].
- Nephrogenic adenoma is more commonly found in the urinary bladder neck and adjacent urethra [7].

#### 3.1.5. Aetiology

- There is an increased incidence of nephrogenic adenoma pursuant to organ transplantation and immunosuppression [7].
- It has been stated that in renal transplant recipients nephrogenic adenoma is derived from exfoliated and implanted renal tubular cells in the urinary tract [9].
- It has also been stated that in other patients nephrogenic adenoma would appear to be metaplastic and not a neoplasm [7].
- Nephrogenic adenoma has been stated to be associated with inflammation [10], intravesical BCG instillations [7], calculi [7], chronic catheterization [7], exstrophy [7], interstitial cystitis [7], intravesical thiotepa instillations [7], malakoplakia [7], Mullerian lesions [7], and surgery (in adults prostatic lesions, in children congenital lesion) [7]. It has also been stated that these aforementioned conditions have been associated with nephrogenic adenoma which are also associated with cystitis glandularis and cystitis cystica [7].

#### 3.1.6. Clinical Presentation

- Nephrogenic adenoma of the urinary bladder tends to present with irritative bladder symptoms including urinary frequency and urgency [7].
- Nephrogenic adenoma of the urinary bladder rarely presents with haematuria [7].
- At cystoscopy, a velvety appearance of the vesical mucosa may be seen and the appearances may quite often be mistaken for papillary urothelial carcinoma by the appearance of the lesion [7].
- It had been stated in the past that nephrogenic adenoma of the urinary bladder exhibits a benign biological behaviour with no malignant transformation even in the presence of significant cytologic atypia [11]; however, subsequently malignant transformation has been reported [12].

#### 3.1.7. Macroscopic Features

Gross examination of specimens of nephrogenic adenoma of the urinary bladder may reveal

- polypoid lesion [7];
- sessile or papillary lesion [7];
- 20% of the lesions which are multiple [7].

#### 3.1.8. Microscopic Features

**Figure 1:**
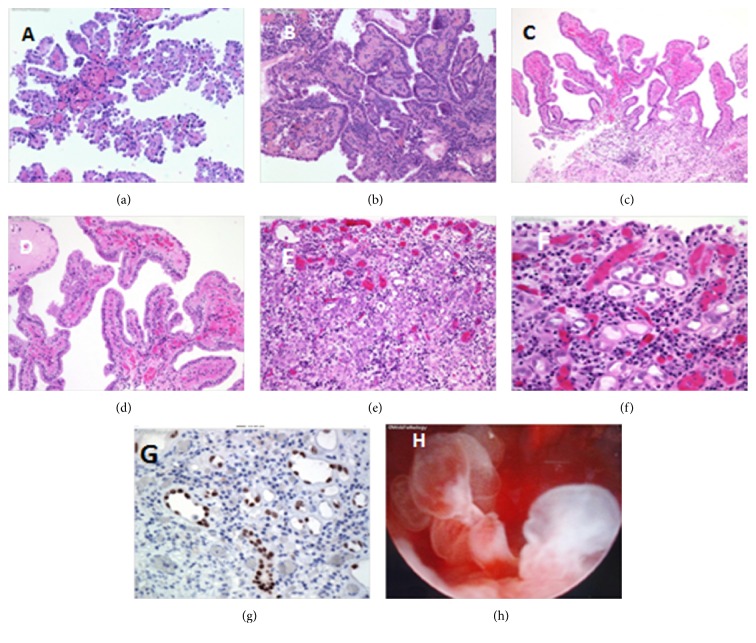
(a) Comments: nephrogenic adenoma is a benign proliferative glandular lesion usually found in the urinary bladder. Bladder calculi, instrumentation, radiation therapy, repeated urinary tract infections, and trauma are the usual predisposing factors. A small percentage of cases arise following renal transplantation. Nephrogenic adenoma displays papillary, polypoid, tubular, or flat configuration and is lined by cuboidal or low columnar epithelium. (b) Comments: nephrogenic adenoma was an incidental finding in this bladder biopsy from a 77-year-old female with a history of urothelial carcinoma of the renal pelvis. The lesion shows tubulopapillary structures lined by cuboidal epithelium with chronic inflammatory cells in the stroma. Nine months later, she was found to have a suspicious “red velvety” area on follow-up cystoscopy. Biopsy showed recurrent nephrogenic adenoma. (c) Comments: nephrogenic adenoma is composed largely of papillary structures arising in the urinary bladder of a patient with history of calculi. A few small tubules are present at the base of the lesion. (d) Comments: high power view of previous image showing nephrogenic adenoma. The nuclei show an orderly arrangement along the surface. The stroma contains chronic inflammatory cells. (e) Comments: nephrogenic adenoma is composed of numerous small as well as cystically dilated tubules. The tubules are lined by plump vesicular nuclei with punctate nucleoli. Some of the tubules have hobnail appearance. Mitotic activity is not increased. (f) Comments: high power view of the previous image showing hobnail nuclei in some of the tubules of nephrogenic adenoma. The nuclei are hyperchromatic but the chromatin is smudged. Punctate nucleoli can be seen. The stroma contains abundant neutrophils, lymphocytes, and plasma cells. The following features are usually not found in nephrogenic adenomas: increased mitotic activity, significant cytologic atypia, solid areas, and deeply infiltrative pattern. (g) Comments: a small percentage of nephrogenic adenomas arise in renal transplant recipients. They most likely develop from renal tubular epithelial cells shed from the transplanted kidney. The immunoreactivity of nephrogenic adenomas for PAX2 and PAX8 markers (shown here) seems to support this hypothesis. PAX2 and PAX8 are transcription factors in the paired box (PAX) gene family. Both are expressed in normal renal tubular epithelium and in lesions derived from them. (h) Comments: cystoscopic view of nephrogenic adenoma in the bladder neck region of a 20-year-old male who presented with history of pelvic pain. The lesion consists of papillary and polypoid edematous-appearing structures. These figures have been reprinted from http://webpathology.com/ with permission from http://webpathology.com/: this permission is exclusive to this request specifically for this paper. Additional usage of any printed or electronic material for which http://webpathology.com/ holds would require copyright permission from http://webpathology.com/.

**Figure 2:**
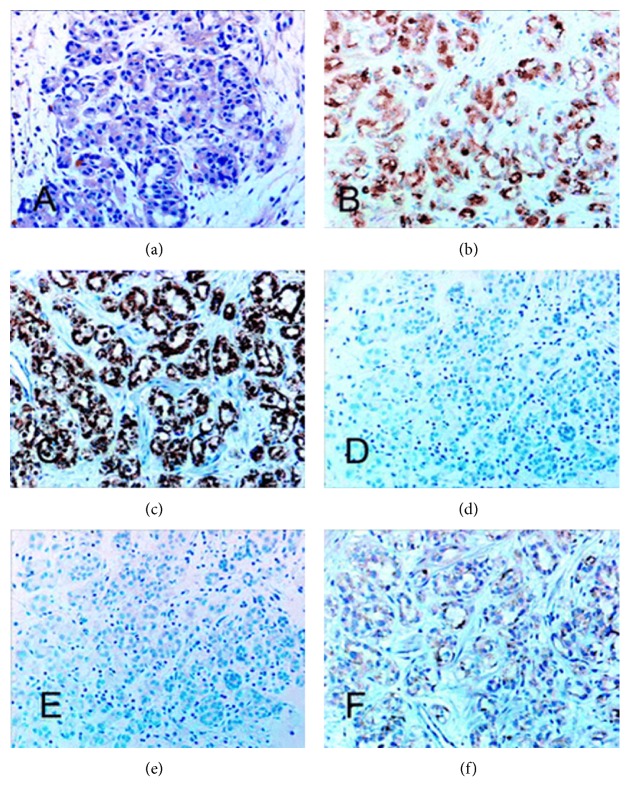
(a, b, c, d, and e) Various stains. Histologic findings and immunoreactivity in a representative case of nephrogenic adenoma. (a) Nephrogenic adenoma with haematoxylin-eosin stain. (b) Positive staining with P504S. (c) Positive staining with epithelial membrane antigen. (d) Negative staining with prostate-specific antigen. (e) Negative staining with CD10. (f) Negative staining with P63 (original magnification ×100) reprinted from [6] Xiao et al. Nephrogenic adenoma: immunohistochemical evaluation for its etiology and differentiation from prostatic adenocarcinoma. Archives of Pathology and Laboratory Medicine, 2006; 130(6): 805–810 with permission from Archives of Pathology and Laboratory Medicine copyright 2006, College of American Pathologists. This permission is exclusive to this request. Additional usage of any printed or electronic material for which the Archives of Pathology and Laboratory Medicine owns the copyright would require permission from the editorial office.

A number of microscopic examination findings from specimens of nephrogenic adenoma of the urinary bladder have been illustrated in Figures 1(a), 1(b), 1(c), 1(d), 1(e), 1(f), 1(g), 1(h), and 2(a). Some of these figures also illustrate the immunohistochemistry feature as well as cystoscopy finding (Figure 1(h)) in a case of nephrogenic adenoma of bladder [13]: On the whole microscopic examination of specimens of nephrogenic adenoma of the urinary bladder tends to reveal.

- Small hollow tubules which look similar to mesonephric tubules are shown, and usually they are lined by a single layer of bland cuboidal or hobnail cells which surround eosinophilic or basophilic secretions [14].
- The cells tend to have clear/eosinophilic cytoplasm, small nuclei, and no prominent nucleoli [7].
- They may have thickened basement membrane [7].
- Usually there is evidence of inflammatory infiltrate (plasma cells and lymphocytes) and stromal oedema [7].
- There is involvement of the lamina propria but the muscularis propria is spared [7].
- The majority of cases also exhibit a cystic pattern and occasionally they are pseudoinfiltrative; they may contain less than ten percent clear cells; they may have small slender papillary structures on the mucosal surface [7].
- Rarely, luminal blue mucin compresses the nuclei which give a signet ring-like appearance [7].
- Presence of minimal atypia and minimal mitotic figures [7].
- There is no evidence of necrosis and no desmoplasia [7].
- With regard to the fibromyxoid subtype of nephrogenic adenoma there is evidence of compressed spindled cells within a fibromyxoid background with only rare tubular and cord-like structures which mimics mucinous carcinoma [14].
- Prostatic urethra nephrogenic adenoma lesions closely resemble adenocarcinoma of prostate and they are AMACR+ [7].

#### 3.1.9. Cytology Characteristics

The cytological characteristics of nephrogenic adenoma of the urinary bladder include the following.

- There are benign features and PAX2+ [15].
- Small clusters and single scattered cells which have central nuclei and vacuolated cytoplasm are seen [7].
- The nuclei exhibit evenly distributed chromatin with small nucleoli and regular nuclear membranes [7].
- Small pseudopapillary clusters of cells with slightly irregular nuclear membranes prominent nucleoli may occasionally be seen [7].
- There may be a background of reactive urothelial cells and squamous cells [16].

#### 3.1.10. Immunohistochemical Staining

See Figures 1(g), 2(b), 2(c), 2(d), and 2(e) for examples.

#### 3.1.11. Positive Stains

Nephrogenic adenoma of the urinary bladder stains positively with the ensuing

- AE1/AE3 (pancytokeratin), CAM5.2, CK7, CK20, and EMA [7];
- CA-125, PAX8, and PAX2 (89% to 100%) [17];
- weak positive staining with PSA or PAP (33%), variable positivity with P504S [18];
- luminal mucin is PAS positive and mucicarmine positive [7].

#### 3.1.12. Negative Stains

Nephrogenic adenoma of the urinary bladder stains negatively with

- CK903, p63, and CD10 (may be focally positive) [9, 19].

#### 3.1.13. Electron Microscopy Features

- It has been stated that the electron microscopic appearance of nephrogenic adenoma of the urinary bladder resembles proximal convoluted tubules [20].

#### 3.1.14. Molecular/Cytogenetics Description

- Monosomy 9 and trisomy 7 have been associated with nephrogenic adenoma of the urinary bladder [21].

#### 3.1.15. Differential Diagnoses

Some of the differential diagnoses of nephrogenic adenoma of the urinary bladder include the following.

- Clear cell adenocarcinoma (CCA): CCA usually occurs in women; it lacks clinical characteristics of adenoma; CCAs tend to be very large tumours; microscopically CCAs exhibit mostly clear cells; there is marked atypia in CCAs; there is muscularis propria invasion in CCA; there is additionally high mitotic rate in CCA; there is necrosis in CCA; there is high Ki-67 percentage in CCA; in CCA, usually PAX2 is negative (even though distinction is usually made upon morphologic grounds); CCA exhibits strong staining with p63 [22–24].
- Endocervicosis may resemble mucinous variant of nephrogenic adenoma [7].
- Papillary urothelial carcinoma is another differential diagnosis which may be mistaken for nephrogenic adenoma but in papillary urothelial carcinoma there is more than 1 layer of urothelial-type cells with atypia [7].
- Prostatic adenocarcinoma of bladder may also mimic nephrogenic adenoma of bladder but in prostatic adenocarcinoma of bladder there is more atypia and strong positive immunohistochemical staining with PSA [7].
- Nested variant of urothelial carcinoma of the bladder may resemble nephrogenic adenoma of the bladder but characteristically in nested variant of urothelial carcinoma there is cystic degeneration of nests and not a single layer and there is marked atypia [7].

#### 3.1.16. Treatment

- The treatment of nephrogenic adenoma of the urinary bladder is resection of the lesion from the bladder but the lesion may recur in view of this long-term follow-up which has been recommended [7].
- There has been a report of a 12-year-old boy with nephrogenic adenoma of the urinary bladder, who was treated with sodium hyaluronate [25].
- Nephrogenic adenoma of the bladder may regress if the associated underlying cause is removed [7].
- In view of the rare reported case of malignant transformation long-term follow-up with cystoscopy would be recommended.

### 3.2. Miscellaneous Narrations from Reported Cases

Vemulakonda et al. [26] reported a case of recurrent nephrogenic adenoma of the bladder in a 10-year-old boy with a history of prune belly syndrome. They stated in their opinion that their reported case was the first reported case of recurrent nephrogenic adenoma. Whilst most cases of nephrogenic adenoma of the urinary bladder have a benign and nonrecurrent biological behaviour the lesson learnt from this report would indicate that in order not to miss a recurrent nephrogenic adenoma of the bladder follow-up cystoscopies would be required.

Hungerhuber et al. [27] reported a 25-year-old man who had had a traumatic urinary bladder which was caused in a car accident. After he had recovered from the accident he developed nephrogenic adenoma and recurrent urinary tract infections. He presented with nephrogenic adenoma of the urinary bladder 18 months pursuant to the accident. The adenoma was treated repeatedly by means of transurethral resections. The initial pathological findings were benign; nevertheless, the last resection revealed that the previous benign adenoma had transformed into a moderately differentiated adenocarcinoma of the bladder (the tumour was present but there was no invasion; the tumour was multifocal; there was no evidence of lymph node invasion; there was no evidence of metastasis; the tumour was moderately differentiated and thus it was classified/reported as grade 2 tumour). He subsequently underwent radical cystectomy and had remained tumour-free for 4 years at the time of publication of the report. This case report would confirm that nephrogenic adenoma of the urinary bladder can recur many times in a benign form and furthermore nephrogenic adenoma of the urinary bladder may subsequently undergo malignant transformation in view of this long-term cystoscopy surveillance which would be necessary in order to detect early all recurrences. It is therefore important for clinicians to be aware of this rare benign tumour's potential to undergo malignant transformation.

Scelzi et al. [28] stated that nephrogenic adenoma is an infrequent benign lesion in the urinary system which occurs in patients who have a history of genitourinary surgery, stone disease, trauma, chronic urinary tract infection, and renal transplantation. They reported the first case of nephrogenic adenoma of the urinary bladder in a 53-year-old man who had a 5-year history of ibuprofen abuse for chronic arthritis. Scelzi et al. [28] stressed the importance of investigating the ibuprofen analgesic abuse for nephrogenic adenoma if nonvisible haematuria and/or irritating lower urinary tract symptoms are present. This report should remind clinicians of the possibility of patients who continuously take ibuprofen subsequently developing nephrogenic adenoma of the urinary bladder.

Campobasso et al. [25] reported the case of a 12-year-old boy with diffuse calculus-producing nephrogenic adenoma in the urinary bladder which was successfully treated with sodium hyaluronate. They stated that

- nephrogenic adenoma is a rare benign lesion of the bladder which occurs as an epithelial response to chronic infection or trauma, probably arising from nephrogenic metaplasia;
- in contrast to nephrogenic adenoma in adults, who present with tumour in the entire ureteral tract, it has been observed exclusively in the urinary bladder in children thus far by the time of publication of their paper.

Even though the main form of treatment for nephrogenic adenoma of the urinary bladder is by means of resection of the lesions, this anecdotal report would suggest that sodium hyaluronate may be an alternative form of treatment and perhaps this treatment option could be used as another option for the management of patients who are not fit to undergo surgical resection of the lesion. Furthermore in view of the fact that only one case has so far been reported it would be a good idea if a multicentre trial is done globally to confirm whether or not hyaluronate is an effective option for the management of nephrogenic adenoma of the urinary bladder.

Hansel et al. [29] stated that nephrogenic adenomas demonstrate various morphological patterns which may rarely be confused with malignant processes, including urothelial and prostatic carcinoma. Hansel et al. [29] described a series of 8 cases of nephrogenic adenoma which contained an admixture of the classic tubular form of nephrogenic adenoma and an unusual spindled and fibromyxoid form of nephrogenic adenoma that closely mimics infiltrating carcinoma. They reported the following.

(i) In all cases the classic tubular form of nephrogenic adenoma composed only a small proportion of the lesion, whereas the remainder consisted of compressed spindled cells within a fibromyxoid background, with only rare tubular and cord-like structures.
(ii) Close examination revealed minimal nuclear atypia in 2 cases, which included small, pinpoint nucleoli and nuclear pseudoinclusions.
(iii) All of the 8 patients were elderly men who had a poor or concurrent history of acinar prostate cancer (*n* = 4), combined acinar prostate and urothelial carcinoma (*n* = 1), urothelial-type adenocarcinoma of the prostate (*n* = 1), urinary bladder urothelial carcinoma (*n* = 1), or no prior reported prostatic or urothelial abnormalities (*n* = 1).
(iv) Five patients had received prior radiotherapy, 1 patient received intravesical instillation of mitomycin-C, and 1 patient also received Bacillus Calmette-Guérin.
(v) The epithelial component of the lesions was positive in all cases for pancytokeratin (AE1/3) and racemase and demonstrated variable cuff of type IV collagen surrounding the tubules. PAX2 was positive with variable extent of labelling. Immunohistochemical staining for prostate-specific antigen was negative. Histochemical stains identified some of the background matrix as mucin, with intense staining for periodic acid-Schiff and focal staining for mucicarmine. Stains for reticulin and amyloid (Congo red stain) and immunohistochemistry for Tamm-Horsfall protein were negative.
(vi) Their case series was the first report of a fibromyxoid subtype of nephrogenic adenoma. Awareness of the entity and the use of ancillary techniques can aid in the diagnosis of this unusual form of nephrogenic adenoma.

Nephrogenic adenoma was first described by Davis [30]. The terminology of nephrogenic adenoma was introduced by Friedman and Kuhlenbeck [31].

The treatment of choice in small nephrogenic adenomas of the urinary bladder is transurethral resection; nevertheless, a high recurrence rate of 37% to 88% had been documented [32]. Rarely, these rare tumours have been reported to be associated with urothelial neoplasms, adenocarcinoma, or squamous cell carcinoma of the urinary bladder [32–34].

It has been stated that the majority of patients with nephrogenic adenoma of the urinary bladder are adults with an increasing incidence in patients who had undergone renal transplantation [35, 36]. Mazal et al. [9] detected the origin of the nephrogenic adenoma in these patients by means of genetic analysis.

It has been stated that approximately 55% of nephrogenic adenomas of the bladder occur in a papillary growth pattern, whilst 35% are sessile and 10% are polypoid [37].

Even though some authors [30, 31] have considered nephrogenic adenomas to be benign lesions, Schultz et al. [38] reported malignant transformation in nephrogenic adenoma which would suggest that nephrogenic adenoma is a premalignant disease, especially in patients who are immunocompromised. Bannowsky et al. [39] were of the opinion that the malignant entity of nephrogenic adenoma is the so-called mesonephroid adenocarcinoma of the urinary bladder; nevertheless, Tse et al. [40] reported an association with transitional cell carcinoma.

Tse et al. [40] undertook a retrospective review of 22 cases of nephrogenic adenoma (NA) which were diagnosed between 1989 and 1996 (7 of which were in renal transplant patients). Tse et al. [40] included in their data collection demographic details, predisposing factors, associated urologic pathology, mode of presentation, cystoscopic finding, management, and follow-up. With regard to the results, Tse et al. [40] reported the following.

- There was a 3 : 1 predominance of men.
- The mean follow-up was 21.4 months (range of 3 months to 50 months).
- Six patients (27%) had one or more recurrences.
- All 22 patients had some form of previous urinary bladder insult or surgery which included recurrent urinary tract infections, urinary tract instrumentation, placements of ureteric stents, cystodiathermy, and open bladder surgery.
- Six cases (27%) were associated with transitional cell carcinoma (TCC) of the urinary bladder, of which 4 had nephrogenic adenoma (NA) lesions directly over or close to the site of previous fulguration.
- In 4 patients, there was a temporal relationship between the administration of intravesical doxorubicin hydrochloride or Bacillus Calmette-Guérin (BCG) and the onset of nephrogenic adenoma (NA) lesions.
- One case was associated with an inverted papilloma that had not been described before.
- In 7 renal transplant cases, 3 lesions were found contralateral to the side of the ureterovesical anastomosis.
- All 22 cases were benign histologically, but one nephrogenic adenoma (NA) was found within a low-grade transitional cell carcinoma (TCC).
- Nineteen cases were followed up regularly with no malignant transformation and three patients were lost to follow-up.

Tse et al. [40] concluded the following.

- Their study had demonstrated an association between nephrogenic adenoma (NA) and urinary bladder cancer.
- Patients with NA, especially those who had been treated with intravesical chemotherapy or BCG, should have regular cystoscopies.
- Fulguration or transurethral resection would appear to be sufficient treatment.
- No renal transplant patient had vesical TCC and NA simultaneously.
- Neither immunosuppression nor ureterovesical anastomosis appeared to be a significant predisposing factor in the transplant patients.

Dow and Young Jr. [41] in 1968 reported the first mesonephroid adenocarcinoma and to emphasize the rarity of mesonephroid adenocarcinoma Vemulakonda et al. [26] stated that up to 2008 only 15 cases with mesonephroid adenocarcinoma had been reported in the literature. Young and Scully [23] stated that histologically a tubular growth pattern is pathognomonic for mesonephroid adenocarcinoma. Hartmann et al. [12] evaluated molecular genetic hybridization in a case of mesonephric adenocarcinoma and they postulated clonal evolution of nephrogenic adenoma to clear cell adenocarcinoma. Hartmann et al. [12] stated that

- nephrogenic metaplasia or nephrogenic adenoma of the urinary tract may present a diagnostic challenge in the practice of surgical pathology;
- previous case reports had suggested the possibility of nephrogenic metaplasia progressing to clear cell adenocarcinoma; nevertheless, a malignant potential of nephrogenic metaplasia had not been generally acknowledged.

Hartmann et al. [12] reported a case of a 70-year-old woman who had multiple recurrences of nephrogenic metaplasia of the urinary bladder with the subsequent development of clear cell adenocarcinoma. Hartmann et al. [12] stated that

- immunohistochemical studies helped them to differentiate the two entities;
- the results of molecular studies, particularly comparative genomic hybridization analysis, suggested clonal evolution of nephrogenic metaplasia to clear cell adenocarcinoma in their case.

Chen and Cheng [42] in 2006 reported their clinical experience with nephrogenic adenoma of the urinary bladder. They stated that, between April 1994 and July 2004, eight patients in their institution were diagnosed with nephrogenic adenoma of the urinary bladder of which 3 were men and 5 were women. The mean age of the 8 patients was 49.6 years and the ages ranged between 23 years and 77 years. The mean follow-up of the patients was 56 months. Chen and Cheng [42] analysed multiple predisposing factors and summarized their findings as follows.

- Nephrogenic adenoma was not associated with transitional cell carcinoma in their series.
- All of the patients had recurrent urinary tract infection.
- Urinary tuberculosis was diagnosed in 2 patients.
- Two patients had previously undergone open urological surgery and 4 patients had previously undergone endoscopic urological management. One patient had a history of urolithiasis. Three patients were noted to have had previous long-term urinary catheterization. Two patients had previously received radiotherapy to the pelvis as treatment for carcinoma of the cervix.
- Urinary frequency and microscopic haematuria were found in all of the patients.
- All the nephrogenic adenomas were treated by means of transurethral resection.
- Recurrent nephrogenic adenoma was diagnosed in 3 patients and the median time to disease relapse was 7 months. The recurrent tumours were also treated by means of endoscopic urological management.

Chen and Cheng [42] concluded the following.

- Nephrogenic adenoma is an uncommon benign metaplastic lesion occurring in the urothelium.
- Transurethral resection of nephrogenic adenoma provides a definite diagnosis and relief of symptoms.
- The recurrence rate of nephrogenic adenoma is relatively high; on view of this, careful and long-term follow-up is necessary.

Zougkas et al. [43] reported four patients with nephrogenic adenoma of the urinary bladder. They stated the following:

- Papilloid or polypoid formations were observed at cystoscopy and transurethral resection of the lesions as nephrogenic adenoma of the urinary bladder. The mean follow-up of the patients was 3.5 years.
- Remission of the symptoms was observed after transurethral resection of the lesions in all of the patients.
- Three out of the four patients presented 1 to 7 relapses, while in one case, after 7 nephrogenic adenoma relapses, urothelial carcinoma of the urinary bladder was diagnosed.

They concluded the following.

- Unlike histological features, the clinical-endoscopic characteristics of nephrogenic adenoma are nonspecific.
- Even if nephrogenic adenoma is not definitely considered like a premalignant condition, nephrogenic adenoma should be followed up frequently and for a long time, because of its high recurrence rate.
- The combinations of cytology, flow cytometry, DNA image analysis, and fluorescence in situ hybridisation of bladder washings or voided urine are of high value in monitoring nephrogenic adenoma of the urothelium.

Porcaro et al. [44] stated that, between September 1976 and June 1999, nephrogenic adenoma of the urinary bladder was diagnosed in 8 patients in their institution, 6 men and 2 women with a 3 : 1 male to female ratio. The ages of the patients ranged from 26 to 80 years and the mean age of the patients was 58.3 years. The follow-up of the patients ranged from 4 months to 194 months and the mean follow-up was 93.5 months. Zougkas et al. [43] summarized their results as follows.

- Nephrogenic adenoma was associated with transitional cell carcinoma in 3 cases.
- Predisposing factors were assessed in all and they found that 5 cases had previous surgery of the lower urinary tract (ureterocystoneostomy in 2 patients, partial cystectomy in 1 patient, repair of vesicocutaneous fistula in 1 patient, multiple urethroplasties in 1 patient), previous endoscopic treatments were undertaken in 2 patients (transurethral resection of prostate in 1 patient and transurethral resection of the vesicle in the other), and a history of intravesical instillation of Bacillus Calmette-Gúerin was assessed in one case.
- Six patients complained of irritating voiding symptoms and 2 patients had haematuria.
- At endoscopy, the lesions appeared polypoid and multifocal in 5 patients and flat and single in 3 patients.
- The lesions were removed via endoscopy which provided relief of symptoms in all of the patients.
- Histopathological examination was used to assess the diagnosis of nephrogenic adenoma, revealing focal atypical cells in one case only.
- Five patients (63%) relapsed 2 to 24 months after management. The recurrences were also treated endoscopic resections.

Porcaro et al. [44] made the following conclusions.

- The clinical and endoscopic features of nephrogenic adenoma of the urinary bladder are not specific, simulating urothelial carcinoma or chronic cystitis.
- Endoscopic management allows accurate histological diagnosis and provides long-lasting relief of symptoms.
- Nephrogenic adenoma needs long-term follow-up, in view of the high risk recurrences and the potential neoplastic degeneration of the metaplastic urothelium.

Martínez-Sanchíz et al. [45] reported 2 cases of nephrogenic adenoma of the urinary bladder with a history of transurethral resection of the bladder and the prostate and a history of prolonged voiding symptoms. In both cases, the findings of encysted tubular structures lined with flattened cuboidal cells without atypia were consistent with the diagnosis of nephrogenic adenoma of the urinary bladder. The two cases were summarized as follows.

*Case 1*. A 60-year-old man who had a history of chronic renal failure presented with a history of severe voiding symptoms and haematuria. He had been having haemodialysis and had previously had transurethral resection of prostate and a bladder diverticulectomy after transurethral resection of bladder neck. He had an ultrasound scan which revealed a raised intravesical lesion. He underwent cystoscopy which revealed multiple trabeculae and diverticula of the urinary bladder and on the left side a superficial papillary lesion which measured about 3 cm in diameter and which was resected. Histological examination of the specimen showed the presence of focal ducts which were lined with cuboidal epithelium without atypia, located in areas of urothelial denudation. The histopathological findings were reported as compatible with nephrogenic adenoma of the urinary bladder. At the time of the report of the paper, the patient was asymptomatic, had been undergoing regular checkups, and had been waiting for a kidney transplant due to end-stage renal disease. 

*Case 2*. An 80-year-old man presented with haematuria. He had a history of two previous transurethral resections for a bladder tumour and benign prostatic hyperplasia. He had an ultrasound scan which revealed a small lesion on the right side of the urinary bladder. He had cystoscopy which showed a 1 cm lesion in the perimeatic area (around the internal urethral meatus) which was resected. Histological examination of the specimen showed encysted tubular structure which was lined with flattened cuboidal cells and the features were reported to be consistent with nephrogenic adenoma of the urinary bladder. The patient at the time of publication of the paper had been undergoing monitoring and his condition remained well.

Kuzaka et al. [46] reported 3 cases of nephrogenic adenoma of the urinary bladder which were treated in their hospital between February 2011 and December 2012. They stated that all of the 3 patients had undergone previous open surgery. Two patients had had kidney transplantation. Visible haematuria and nonvisible haematuria were found in 2 patients. One patient had recurrent urinary tract infection. One patient had nephrogenic adenoma which was associated transitional cell carcinoma (TCC). The remaining two patients had nephrogenic adenoma of the bladder only. Kuzaka et al. [46] also reported that

- recurrent nephrogenic adenomas were diagnosed in 2 patients and the time to disease relapse was 5 months and 9 months;
- all the nephrogenic adenomas and recurrent tumours were treated by means of transurethral resection.

Kuzaka et al. [46] concluded that

- even though nephrogenic adenoma is a benign metaplastic lesion of the urothelium, its recurrence rate is relatively high; hence careful and regular follow-up is necessary;
- endoscopic characteristics of nephrogenic adenoma are not specific and definite diagnosis must be made after histological analysis of resected specimens.

Filly and Baskin [47] reported a 16-year-old male patient who had visible haematuria and whose ultrasound scan showed multipapillary excrescences in the urinary bladder. When he was aged 4 years, he underwent bilateral reimplantation of ureters for recurrent urinary tract infections and vesicoureteric reflux. The kidneys showed calyceal dilatation of the infundibula or renal pelvis. There were no overlying cortical scars. He subsequently underwent cystoscopy which showed frond-like sessile lesions throughout the posterior and lateral walls of the urinary bladder. He had random biopsies of the bladder lesions and histological examination of the lesions confirmed nephrogenic adenoma as the underlying cause of the ultrasound scan findings. By the time of publication of the paper, he had had a 2-year follow-up during which there were 2 additional sporadic episodes of visible haematuria.

Pierre-Louis et al. [48] reported 2 cases of nephrogenic adenoma of the urinary bladder as follows. 

*Case 1*. A 62-year-old, black man presented with haematuria. He underwent cystoscopy which revealed multiple papillary excrescences covering the right hemitrigone and the posterolateral wall of the urinary bladder. Histological examination of the excised tissue was diagnosed as nephrogenic adenoma. One year prior to his admission he had undergone suprapubic prostatectomy. Subsequently he had repeatedly complained of urinary frequency, urgency, nocturia, and intermittent haematuria. Small fungating masses were found on the lateral wall of the urinary bladder and were partially resected. A pathological diagnosis of cystitis cystica was made. A bladder biopsy which was performed two days prior to admission revealed cystitis glandularis. 

*Case 2*. A 67-year-old, black man presented with respiratory distress. He had undergone right inguinal herniorrhaphy and a transurethral prostatectomy four years preceding his admission. His urinalysis revealed pyuria and haematuria. Several polypoid masses which measured 1/5 cm × 1.2 cm × 1 cm were noted over the posterolateral wall of the urinary bladder. Nephrogenic adenoma of the urinary bladder was diagnosed.

Pierre-Louis et al. [48] reported the pathological findings of the two tumours as follows.

- Histologically, both lesions showed a markedly thickened, bladder mucosal lining and focal polyps. Occasionally, these papillary projections were noted to have complex infoldings. The lamina propria contained numerous tubules and structures that were cystically dilated. The tubular structures resembled the tubules of the developing kidney. The epithelial cells which lined the smaller tubules possessed large, rather atypical nuclei.
- Ultrastructural examination revealed that the small tubular structures were lined by tall, columnar epithelial cells and had irregularly indented nuclei with prominent nucleoli. The cells rested on distinct basal lumens. The extracellular spaces were distended. Rarely, the basal lamina was multilayered. The apical parts of the cells had distinct intercellular junctions, and the luminal surface was covered with short microvilli. The cytoplasm, which was abundant, contained numerous mitochondria, rough endoplasmic reticulum, and sparse lysosomal granules. The basal part of the epithelium exhibited multilayered basal lumens. There were no asymmetric membranes on the luminal surface of these cells.
- Immunohistochemistry examinations using soybean and peanut-lectin agglutinins revealed SBA and, more importantly, free PNA-receptor sites at the luminal surface of the epithelial cells which lined the atypical tubules and the papillary projections.

Pierre-Louis et al. [48] commented that:

- their two cases of nephrogenic adenoma were described based upon light and electron microscopic as well as immunohistochemistry studies;
- their reported patients developed nephrogenic adenoma pursuant to surgical trauma;
- the majority of patients reported by a number of authors [10, 30–32, 49–57] had had previous surgical manipulation, infections, or some trauma to the urinary tract;
- the association of urinary bladder irritation with the development of nephrogenic adenoma would strongly support metaplastic origin of nephrogenic adenoma of the urinary bladder;
- the findings of the ultrastructural studies of their patients' bladder lesions showed an absence of urothelial-cell differentiation such as asymmetric membranes or numerous desmosomes in the nephrogenic adenoma cells;
- the complex basal-intercellular interdigitations are luminal microvilli in nephrogenic adenoma cells resembling the ultrastructural characteristics of renal tubular cells;
- reports from ultrastructural studies of other nephrogenic adenomas show electron microscopic findings that are similar to those in their two reported cases; the tubular structures of those lesions had been described as resembling mesonephric tubules by Molland et al. [54], proximal convoluted tubules as described by Bhagavan et al. [20], Henle's loop and collecting tubules as described by Taneja et al. [56], or tubular structures lined by immature urothelial basal cells as described by a number of authors [53, 57, 58];
- Devine et al. [59] had undertaken a study of the lectin-binding pattern of 19 cases of nephrogenic adenoma and determined some of the cellular-coat sugars in the lesion; the studies of Devine et al. [59] as well as their own study show that the luminal surfaces of cells which line the tubular structures and papillary fronds of nephrogenic adenomas stain for SBA and free surface PNA-receptor sites;
- PNA-receptor sites are not present in normal urothelium as reported by a number of authors [59–63]; they are also not present in cystitis cystica or in squamous metaplasia of the urinary bladder epithelium [59];
- the lectin receptors have been reported to be present on the luminal surface of the mesonephric and the metanephric tubules of human embryos [57] and the distal and collecting tubules of the normal adult kidney [64, 65];
- Some authors [60, 61, 63] had stated that urothelial cells only exhibit PNA receptors on their cellular surface after neuraminidase digestion.

**Figure 3:**
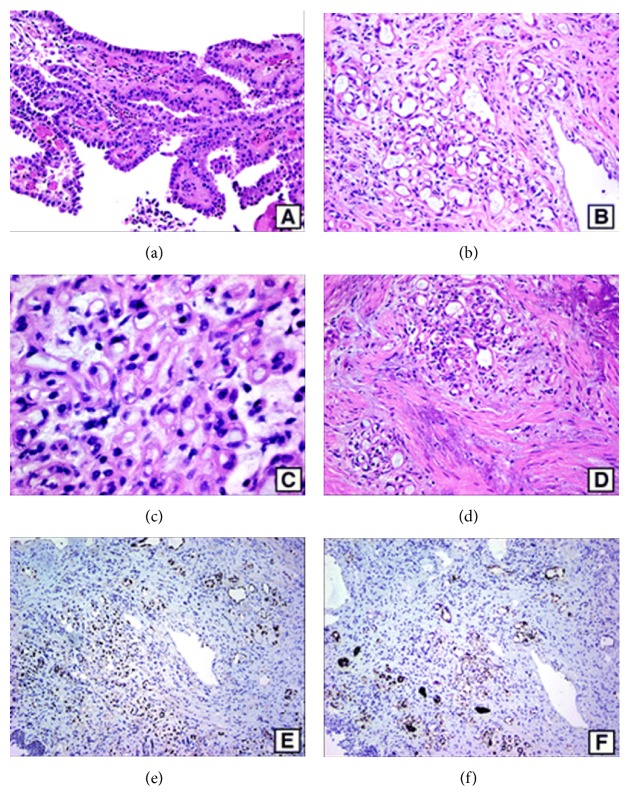
(a) Nephrogenic adenoma with papillary architecture, lined by columnar cells with pink cytoplasm. (b) Nephrogenic adenoma with tubular architecture composed of small tubules lined by low cuboidal to flattened epithelial cells containing blue mucin. Some nuclei show degenerative atypia with smudgy indistinct chromatin. (c) Nephrogenic adenoma with small tubules simulating signet ring cells and degenerative nuclear atypia. (d) Nephrogenic adenoma with invasion into superficial invasion. (e) Nephrogenic adenoma with strong nuclear positivity with PAX2. (f) Nephrogenic adenoma with cytoplasmic positivity with *α*-methylacyl-CoA racemase (AMACR) (haematoxylin-eosin, original magnification ×200 (a through d), PAX2, original magnification ×100 (e); AMACR, original magnification ×100 (f)). The figure was reprinted from Kunju Nephrogenic adenoma of report of a case and review of morphologic mimics. Archives of Pathology and Laboratory Medicine 2010; 134: 1455–1459 with permission from Archives of Pathology and Laboratory Medicine copyright 2008, College of American Pathologists. This permission is exclusive to this request specifically for this paper. Additional usage of any printed or electronic material for which the Archives of Pathology and Laboratory Medicine Copy owns copyright would require permission from the editorial office.

**Figure 4:**
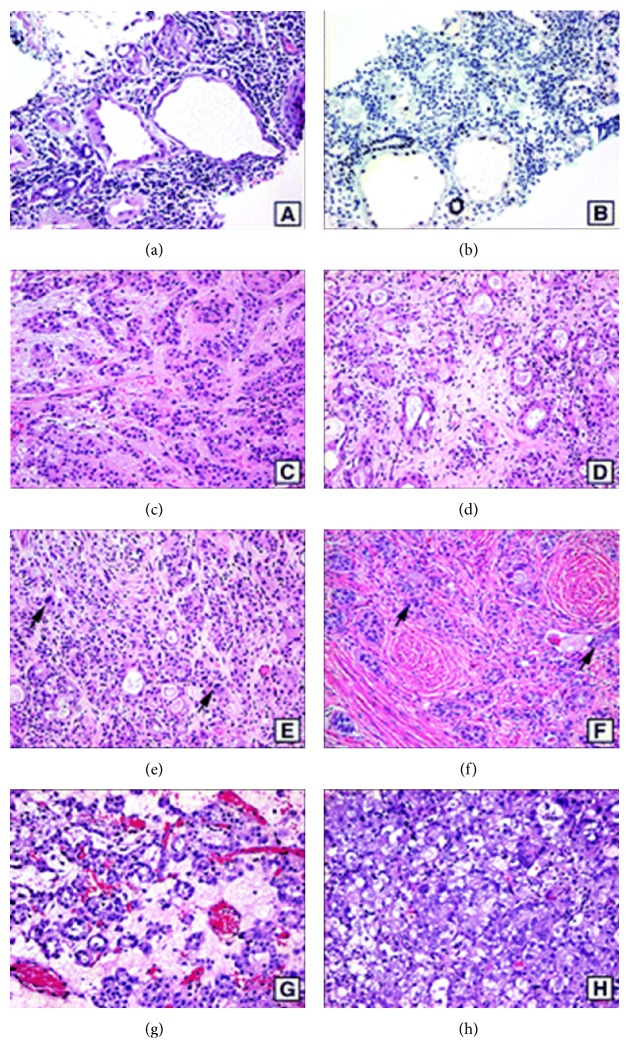
(a) Nephrogenic adenoma (NA) involving prostate urethra. Note tubules and cysts lined by hobnail cells with a hyaline sheath surrounding some of the tubules, inflamed stroma, adjacent urothelium, and no significant cytologic atypia. (b) Nephrogenic adenoma involving prostatic urethra showing nuclear positivity with PAX2; (c through f) nested variant of urothelial carcinoma, (c) irregular proliferation of discrete nests with bland cytology separated by oedematous stroma. (d) Frequent tubular differentiation is noted. (e) Despite overall bland cytology, cells with random cytologic atypia can be seen (arrows). (f) Nested variant of urothelial carcinoma invading into muscularis propria and showing focal high-grade cytologic atypia (arrows). (g) Clear cell adenocarcinoma with prominent tubular architecture mimicking NA. (h) Clear cell adenocarcinoma with solid growth pattern and obvious nuclear atypia (haematoxylin-eosin, original magnification ×200 (a through f and h) and ×100 (g)). The figure was reprinted from Kunju Nephrogenic adenoma of report of a case and review of morphologic mimics. Archives of Pathology and Laboratory Medicine 2010; 134: 1455–1459 with permission from Archives of Pathology and Laboratory Medicine copyright 2008, College of American Pathologists. This permission is exclusive to this request specifically for this paper. Additional usage of any printed or electronic material for which the Archives of Pathology and Laboratory Medicine owns copyright would require permission from the editorial office.

Kunju [2] reported a 71-year-old man who had a previous history of carcinoma of the urinary bladder and who had undergone transurethral resection of bladder tumour twice in the preceding 12 months. His latest transurethral resection specimen had shown a proliferation of numerous small tubular structures which had attenuated hobnail cells, haphazard growth pattern, luminal blue mucin, and mild degenerative nuclear atypia, presence of signet ring-like tubules, infiltrating pattern into the deep lamina propria, including focal involvement of muscularis mucosae and superficial muscularis propria which were considered to be unusual, and hence immunohistochemical studies were undertaken to exclude malignancy. A diagnosis of nephrogenic adenoma of the urinary bladder with some unusual morphological characteristics was made. Kunju [2] stated the following:

- Nephrogenic adenomas may show papillae as well as a focal solid growth pattern; nevertheless, conspicuous diffuse solid growth pattern is extremely uncommon.
- The cells lining tubules, cysts, and papillae are cuboidal to low columnar with scant cytoplasm (Figures 3(a) and 3(b)).
- Occasional clear cells may be seen especially in the solid areas.
- Even though enlarged nuclei and prominent nucleoli may be observed in some cases of nephrogenic adenoma of bladder, significant nuclear atypia, including presence of mitosis, is very rare; nuclear atypia when present appears degenerative with indistinct smudgy chromatin.
- Small tubules with blue mucin lined by a single cell which resemble signet ring cells may be seen (Figure 3(c)). Nephrogenic adenomas typically are not invasive tumours though rare cases of nephrogenic adenoma may focally involve the superficial muscularis propria (Figure 3(d)) [66].
- Nephrogenic adenomas are typically positive with cytokeratin 7 (CK7), *α*-methylacyl-CoA racemase (AMACR), P504S, PAX2, and epithelial membrane antigen and are usually negative with p63 [6, 18] (Figures 3(e) and 3(f)).
- PAX2, which is a key renal transcription factor, is commonly expressed in nephrogenic adenoma that some authors [9] had interpreted as evidence in support of renal tubular origin of nephrogenic adenoma.
- Nephrogenic adenomas exhibit patchy staining with high-molecular weight cytokeratin (34*β*E12), even though a subset of these (40% to 45%) may be completely negative [18, 67].
- The histological characteristics that favour nephrogenic adenoma of the prostatic urethra from adenocarcinoma of the prostate include lack of significant cytologic atypia and mitosis and presence of adjacent urothelium with papillary architecture and vascular-like cystic tubules which are lined by hobnail cells that are set in oedematous and inflammatory stroma (Figure 4(a)).
- Cytokeratin 7 and PAX2 positivity of nephrogenic adenoma (Figure 4(b)) can be useful to differentiate nephrogenic adenoma from adenocarcinoma of prostate, which is negative with these markers.
- Nested variant of urothelial carcinoma (Figures 4(c) to 4(f)) exhibits distinctive patterns in the superficial and deep portions of the tumour. In biopsies and transurethral resection specimens, the superficial component of nested variant of urothelial carcinoma which is a differential diagnosis of nephrogenic adenoma, is characterized by small, crowded, tightly packed nests with focal tubular differentiation that could be prominent in some cases. The nests commonly possess central lumens which resemble cystitis cystica [68–70]. The majority of the nests have a deceptively bland cytology within the superficial areas of the tumour, though random cytologic atypia within nests is commonly observed. Within the deeper parts, a prominent infiltrative base with frequent muscularis propria invasion and focal high-grade cytologic atypia is noted (Figures 4(e) and 4(f)).
- Another rare entity which may mimic nephrogenic adenoma of the urinary bladder is clear cell adenocarcinoma (Figures 4(g) and 4(h)); this tumour may show foci with tubular, cystic, and papillary architecture lacking significant cytologic atypia, focally resembling nephrogenic adenoma.

Safaei et al. [71] reported a 55-year-old woman with urinary problem. She underwent cystoscopy which revealed a sessile mass which was resected. Histology of the specimen revealed circumscribed proliferation of tubules, cysts, and papillae which were lined by low cuboidal to columnar epithelial cells. Immunohistochemical staining of the tumour was strongly positive for CK7, P504S, CD10, and EMA but negative for CK20, PSA, and P63. A diagnosis of nephrogenic adenoma of urinary bladder was made. Her urinary symptoms improved after resection of the lesion. She was lost to follow-up after 5 months of follow-up.

## 4. Conclusions

Nephrogenic adenoma of the urinary bladder (NAUB) is a peculiar lesion of the urinary bladder which is characterized partly by villous structures and partly by gland-like lesions.

The clinical presentation and endoscopic characteristics of NAUB are nonspecific.

Diagnosis of NAUB can be established with the help of microscopic and immunohistochemical staining characteristics of the tumour and also with the help of cytology and electron microscopic features of the tumour.

Combinations of cytology, flow cytometry, DNA image analysis, and fluorescence in situ hybridization of bladder washings and voided urine are useful in the monitoring of NAUB.

NAUB is not considered malignant but it is associated with high recurrence rate. In view of this following the initial treatment for NAUB a long-term follow-up by cystoscopic examination should be undertaken in order to identify and treat recurrent tumours and to identify early the few recurrent tumours that may undergo malignant transformation.

There is no consensus opinion regarding the best treatment option that would avoid or reduce recurrence of NAUB following initial treatment and considering the fact that intravesical therapy has been documented to be associated with NAUB there is need for a global multicentre trial to identify the best treatment option that would help reduce and avoid subsequent recurrences.
